# Comparison of Vonoprazan and Low-Dose Amoxicillin Dual Therapy with Bismuth-Containing Quadruple Therapy for Naïve *Helicobacter pylori* Eradication: A Single-Center, Open-Label, Randomized Control Trial

**DOI:** 10.3390/antibiotics14100990

**Published:** 2025-10-03

**Authors:** Xue Fan, Yanyan Shi, Yuan Li, Xiangchun Lin

**Affiliations:** 1 Department of Gastroenterology, Peking University International Hospital, Beijing 102206, China; fanxue@pkuih.edu.cn (X.F.); liyuan5@pkuih.edu.cn (Y.L.); 2 Research Center of Clinical Epidemiology, Peking University Third Hospital, Beijing 100191, China; shiyanyan@bjmu.edu.cn

**Keywords:** *Helicobacter pylori*, vonoprazan, proton pump inhibitors, amoxicillin, dual therapy, quadruple therapy

## Abstract

Objective: This study aimed to compare the effectiveness and safety of vonoprazan–amoxicillin (VA) dual therapy with modified bismuth-containing quadruple therapy (esomeprazole, bismuth, amoxicillin, and clarithromycin; EBAC) in treatment-naïve patients infected with *Helicobacter pylori* (*H. pylori*). Methods: In this single-center, open-label, randomized controlled trial conducted from July to December 2024, a total of 504 *H. pylori*-positive patients were randomly allocated to receive either VA (vonoprazan 20 mg and amoxicillin 1000 mg, twice daily for 14 days) or EBAC (esomeprazole 20 mg bid, bismuth potassium citrate 220 mg bid, amoxicillin 1000 mg bid, clarithromycin 500 mg bid, twice daily for 14 days). The primary endpoint was the *H. pylori* eradication rate, and the secondary endpoint was safety. Results: In the intention-to-treat (ITT) analysis, the eradication rates were 79.4% (200/252) in the VA group and 85.7% (216/252) in the EBAC group (*p* = 0.060). Per-protocol (PP) analysis showed comparable eradication rates between the two groups (92.1% [197/214] vs. 93.0% [213/229], *p* = 0.712), confirming the non-inferiority of VA compared to EBAC. The incidence of adverse events was significantly fewer in the VA group (27.2% vs. 42.7%, *p* < 0.001). Logistic regression identified medication adherence (≥80%) as the only independent predictor of successful eradication (OR 17.557, *p* < 0.001). Conclusions: VA dual therapy achieved comparable *H. pylori* eradication rates to EBAC, while offering better safety and a more convenient regimen, supporting it as a preferred first-line treatment for *H. pylori* infection.

## 1. Introduction

*Helicobacter pylori* (*H. pylori*) was first observed in gastric mucosa in the 19th century but its connection with gastritis was not established until around 40 years ago [1]. *H. pylori* is strongly associated with a range of gastrointestinal diseases, including up to 90% of duodenal ulcers, 80% of gastric ulcers, mucosa-associated lymphoid tissue lymphoma and gastric cancer. In 2014, the World Health Organization classified *H. pylori* as a high-risk factor for gastric cancer and, in 2017, recommended its eradication as a global strategy to reduce gastric cancer mortality. Numerous studies have shown that *H. pylori* eradication contributes significantly to the prevention and treatment of gastrointestinal diseases by reducing gastric cancer incidence and preventing the recurrence of peptic ulcers. Recent studies also indicate a steady decrease in *H. pylori* prevalence worldwide [2].

In most countries, proton pump inhibitor (PPI)-based triple therapy and bismuth-containing quadruple therapy are currently recommended as first-line treatment regimens for *H. pylori* eradication [3,4,5,6,7]. However, *H. pylori* eradication rates have gradually declined over time, and suboptimal acid suppression remains a key factor in treatment failure. Adequate and sustained gastric acid inhibition is pivotal for maintaining *H. pylori* in a replicative, drug-sensitive state and stabilizing acid-labile antibiotics, such as clarithromycin and amoxicillin. PPIs, however, which are affected by food intake and CYP2C19 genetic polymorphisms, have limitations such as delayed onset of action and short half-life, potentially reducing treatment efficacy.

Achieving successful *H. pylori* eradication faces two major challenges, insufficient acid suppression and increasing antibiotic resistance. Furthermore, conventional quadruple therapy is complicated by a multi-drug regimen and frequent adverse events, which may affect patient compliance. Vonoprazan, a novel reversible H^+^/K^+^-ATPase inhibitor, provides faster onset of action, more potent and prolonged acid suppression, and is unaffected by food intake or CYP2C19 polymorphisms. These pharmacological advantages make it a promising alternative for enhancing *H. pylori* eradication efficacy. Vonoprazan was approved for the treatment of *H. pylori* infection in 2023. Although several meta-analyses [8,9,10] suggest that vonoprazan-based regimens are more effective than conventional PPI-based therapies, further clinical data are needed to confirm the efficacy and safety of vonoprazan-based *H. pylori* eradication strategies.

The objective of this study was to evaluate the efficacy, safety, and treatment compliance of a vonoprazan–amoxicillin dual therapy regimen in treatment-naïve patients with *H. pylori* infection.

## 2. Methods

### 2.1. Study Design and Participants

This was a single-center, open-label, randomized, controlled clinical trial conducted in China, approved by the Institutional Review Board of Peking University International Hospital (Approval No. 2024-KY-0037-01). The trial adhered to the Declaration of Helsinki and the International Council for Harmonisation Good Clinical Practice guidelines, and was registered at chictr.org.cn (ChiCTR2500098029). Written informed consent was obtained from all participants prior to study enrollment. All co-authors had full data access and approved the final manuscript.

Eligible patients were adults aged 18 to 70 years, with no prior treatment for *H. pylori* infection and who provided consent to participate. Diagnosis of *H. pylori* infection was confirmed by at least one of the following methods: gastric biopsy using histochemical staining, tissue culture, the ^14^C- or/and ^13^C-urea breath test (UBT). Exclusion criteria included patients if they met any of the following conditions: use of antibiotics, bismuth compounds, or traditional Chinese medicines with antimicrobial properties within the past month, or use of PPIs, H_2_-receptor antagonists, or other drugs affecting *H. pylori* activity within the past 2 weeks; presence of severe underlying medical conditions (e.g., hepatic, pulmonary, or cardiovascular diseases); allergy to penicillin or any medications used in the trial; pregnancy or lactation; severe gastrointestinal disorders, including malignancy, gastrointestinal bleeding, Zollinger–Ellison syndrome, or history of subtotal gastrectomy; and any other condition compromising patient safety or adherence deemed by the investigators.

### 2.2. Trial Assessments

Demographic and clinical characteristics were gathered at screening. Treatment- emergent adverse events (TEAEs) and concomitant medications were documented throughout the study for all patients who received at least one dose of the study drug.

The primary endpoint was the *H. pylori* eradication rate, assessed by ^13^C-UBT performed at least 4 weeks after treatment completion.

Secondary endpoints encompassed the incidence and severity of adverse events (AEs), as well as treatment adherence. AE severity was graded as none, mild (transient and well-tolerated; no impact on daily activities), moderate (partially interferes with daily activities) and severe (significantly affects daily life, requires hospitalization, or results in death related to the study). Poor adherence was defined as taking <80% of prescribed medication.

### 2.3. Randomisation and Interventions

At the trial’s onset, participants were randomly assigned (1:1) to the intervention or control group using a computer-generated random number sequence. Allocation was concealed using sequentially numbered, opaque, sealed envelopes prepared by an independent coordinator. Patients received either VA dual therapy, consisting of vonoprazan 20 mg twice a day (20 mg/tablet, Takeda Pharmaceutical, Osaka, Japan) and amoxicillin 1000 mg twice a day (250 mg/tablet, Zhuhai United Laboratories Co., Ltd., Zhuhai, China), or EBAC therapy, consisting of esomeprazole 20 mg twice a day (20 mg/tablet, AstraZeneca, Cambridge, UK), bismuth potassium citrate 220 mg twice a day(110 mg/tablet, Livzon Pharmaceutical, Zhuhai, China), amoxicillin 1000 mg twice a day (250 mg/capsule, Zhuhai United Laboratories Co., Ltd.), and clarithromycin 500 mg twice a day (250 mg/tablet, Abbott, Chicago, IL, USA), each administered for 14 days.

Participants took esomeprazole and bismuth 30 min before meals, while vonoprazan, amoxicillin, and clarithromycin were taken 30 min after meals. Randomization was conducted using a computer-generated sequence. This study was open-labeled, and both patients and physicians knew the treatment received. The technician responsible for performing ^13^C-UBT was blinded. All participants were instructed about the medication regimen, potential AE, and AE reporting procedures.

### 2.4. Sample Size Calculation and Statistical Analysis

This non-inferiority trial was designed as with a non-inferiority margin of −10%, 80% power (1- β), and an one-sided alpha of 0.025. Based on prior trials that assumed an eradication rate of 85% [9,11] for both groups, a minimum of 201 participants per group was determined using PASS 2021 software. To accommodate a potential 20% dropout rate, the final sample size was increased to 252 patients per group.

Three analysis cohorts were defined: (1) intention-to-treat (ITT) cohort: all randomized patients with at least one follow-up evaluation; patients who were lost to follow-up were regarded as treatment failures; (2) modified intention-to-treat (m ITT) cohort: patients who took at least one dose of medication and underwent the ^13^C-UBT ≥ 4 weeks after treatment, irrespective of adherence; and (3) per-protocol (PP) cohort: patients with ≥80% adherence who fulfilled all study procedures.

Continuous variables were reported as mean ± standard deviation (SD), and categorical variables as numbers and percentages (%). Appropriate statistical tests were applied: Student’s *t*-test for continuous variables and the χ^2^ test (chi-square test) or Fisher’s exact test for categorical variables. Univariate and multivariate logistic regression analyses were performed to identify potential factors influencing *H. pylori* eradication rates, based on the ITT population.

All *p*-values were two-tailed except for non-inferiority testing and a *p*-value < 0.05 was considered statistically significant. Statistical analyses were conducted using SPSS version 26.0.

## 3. Results

### 3.1. Baseline Data and Clinical Characteristics of Patients

**Figure 1:**
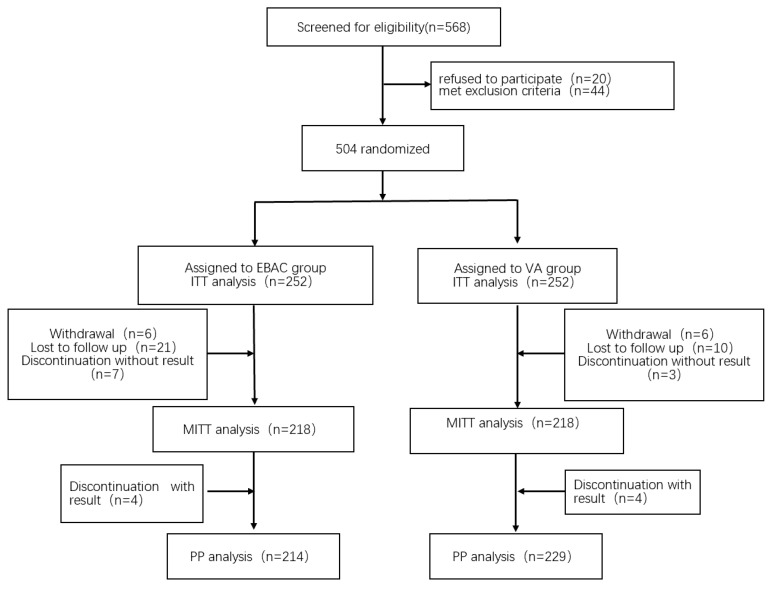
Flow chart of patient grouping. EBAC, esomeprazole, bismuth, amoxicillin, and clarithromycin; VA, vonoprazan, amoxicillin; ITT, intention-to-treat; mITT, modified intention-to-treat; PP, per-protocol.

From July 2024 to January 2025, a comprehensive study was conducted involving 568 patients. Among them, 504 patients were randomly assigned to the two treatment groups in two specific treatment groups, 252 in the EBAC group and 252 in the VA group (Figure 1). The final follow-up was completed in May 2025.

As presented in Table 1, there were no statistically significant differences between the two groups in baseline demographic or clinical characteristics, including gender, age, body mass index (BMI), education level, cigarette smoking or alcohol drinking habits, family history of gastric cancer, endoscopic diagnosis, symptoms or comorbidities.

In the VA dual therapy group, a total of 34 patients either withdrew consent, were lost to follow-up, or discontinued treatment. In the EBAC quadruple therapy group, a total of 19 patients faced similar issues. Due to their lack of ^13^C-UBT results, they were classified as treatment failures in the ITT analysis and excluded from the mITT analysis. Additionally, four discontinued patients in each group (VA and EBAC) who completed the post-treatment ^13^C-UBT assessment failed to meet the protocol-defined adherence criteria, and were excluded from the PP analysis.

### 3.2. Comparison of Eradication Rates

Eradication rates for the VA and EBAC groups via different analysis sets are shown in Table 2.

In the ITT analysis, the eradication rate was 79.4% (200/252; 95% CI: 74.3–84.4%) in the VA group and 85.7% (216/252; 95% CI: 81.4–90.1%) in the EBAC group. The PP analysis showed eradication rates of 92.1% (197/214; 95% CI: 88.4–95.7%) for the VA group and 93.0% (213/229; 95% CI: 89.7–96.3%) for the EBAC group. Similarly, the mITT analysis yielded eradication rates of 91.7% (200/218; 95% CI: 88.1–95.4%) and 92.7% (216/233; 95% CI: 89.3–96.1%) in the VA and EBAC groups, respectively.

The between-group rate differences (RDs) were 6.35% (95% CI: −0.58% to 13.23%) in the ITT analysis, 0.96% (95% CI: −4.39% to 6.45%) in the mITT analysis, and 0.96% (95% CI: −4.36% to 6.42%) in the PP analysis. While the ITT analysis showed the largest point estimate for difference, both the mITT and PP analyses yielded minimal differences with narrower confidence intervals, suggesting that protocol deviations likely influenced the ITT results.

All confidence intervals for RD included zero, suggesting no statistically significant difference between regimens. The *p*-values from the ITT, mITT, and PP analyses were 0.060, 0.714, and 0.712, respectively, confirming that the VA regimen was non-inferior to the EBAC regimen.

### 3.3. Logistic Multivariate Analysis of Factors Affecting Eradication Rate

A logistic regression model was used to assess factors associated with *H. pylori* eradication (Table 3). Variables included age, sex, alcohol use, smoking status, BMI, education, family history of gastric cancer, endoscopic findings, treatment adherence, and treatment group.

Multivariate logistic regression analyses were conducted. In the analysis, good adherence (defined as medication intake ≥ 80%) was strongly associated with successful eradication, with an odds ratio (OR) of 17.557 (95% CI: 5.678–54.287, *p* < 0.001).

Various factors such as age, sex, lifestyle habits, family history, and treatment methods were examined for their impact on eradication success rates. However, none of these variables were found to have a significant correlation with the outcomes of eradication in both the univariate and multivariate analyses.

### 3.4. Adverse Reactions and Patient Compliance

A total of 492 patients were analyzed for safety in a study, with 31 patients lost to follow-up and 12 withdrawals after randomization. Adverse reactions observed during treatment in the EBAC and VA groups are detailed in Table 4. The most commonly reported adverse reactions included bitter taste, diarrhea, epigastric discomfort, nausea, vomiting, and hunger sensation.

No serious adverse events requiring medical intervention or hospitalization were documented in either treatment group. All adverse events were successfully resolved following the completion or discontinuation of therapy. While a few patients experienced a rash, it was effectively managed with medication and did not lead to any fatalities or hospital admissions related to treatment.

The incidence of adverse events was significantly lower in the VA group compared to the EBAC group (27.2% vs. 42.7%, *p* < 0.001). However, rates of discontinuation due to adverse events were low and comparable between groups (0.4% in VA vs. 2.0% in EBAC, *p* = 0.737).

Medication adherence was high in both groups, with 96.3% (237/246) in the VA group and 94.7% (230/246) in the EBAC group achieving good compliance (≥80% medication intake); the difference was not significant (*p* = 0.151).

## 4. Discussion

Globally, *H. pylori* infects approximately 43% of the population, with rates varying from 34.7% in developed countries to 50.8% in developing regions [12,13]. Eradication of *H. pylori* is associated with a reduced risk of gastric cancer, and the observed decline in gastric cancer incidence in some countries has been linked to reduced infection rates. A large-scale survey in China [14], indicated a household infection rate of 71.2%, markedly higher than the individual rate of 40.7%. These results emphasize the necessity for effective, safe, and well-tolerated regimens for eradicating *H. pylori*.

Instead of reiterating the general background, this study particularly highlights the clinical challenge of increasing antibiotic resistance and insufficient acid suppression as the two most important barriers to successful eradication. Recent meta-analyses [15,16] demonstrate a concerning upward trend in overall resistance rates, likely attributable to significant geographic variability. Surveillance data [17] reveal alarming primary resistance rates from 1990 to 2022: clarithromycin (30%), metronidazole (61%), and levofloxacin (35%), while amoxicillin (6%) and tetracycline (4%) maintain relatively stable susceptibility profiles. Striking disparities exist across regions, with developing nations exhibiting approximately 25% higher resistance rates [13,15]—up to 2–3 times those observed in Western countries like the United States, United Kingdom, and Canada [18]. A China-specific meta-analysis [19] urgently calls for enhanced resistance monitoring, particularly for metronidazole, levofloxacin, and clarithromycin. Given that antibiotic resistance remains the dominant cause of eradication failure, the choice of amoxicillin as the backbone of dual therapy is supported by its consistently low resistance rate and favorable safety profile. Inadequate acid suppression represents the other key limitation of conventional regimens. The bactericidal efficacy against *H. pylori* significantly diminishes in low-pH environments. The Maastricht VI Consensus [7] establishes clear therapeutic targets: optimal eradication requires sustained acid suppression with median intragastric pH > 6 or >90% time above pH 4 during 24 h monitoring. Acid suppression directly influences eradication by affecting antibiotic stability and bacterial susceptibility.

Building on these challenges, our study focused on vonoprazan–amoxicillin dual therapy, leveraging vonoprazan’s rapid and sustained acid suppression to improve clinical outcomes while simplifying the regimen compared to traditional multi-drug therapies. To address these challenges, the therapeutic regimens for *H. pylori* eradication have evolved from concomitant therapy, sequential therapy, and bismuth-containing quadruple therapy to the current dual therapy. Since *H. pylori* treatment must account for bacterial susceptibility, amoxicillin has emerged as the primary antibiotic choice for dual therapy due to its low resistance rates, high efficacy, and favorable safety profile. In recent years, high-dose PPI-based dual therapy has shown promise and is increasingly being adopted in clinical practice, though the evidence remains inconsistent. A randomized controlled trial conducted in Taiwan [20] demonstrated that high-dose amoxicillin combined with rabeprazole monotherapy achieved superior ITT eradication rates compared to standard triple therapy (95% vs. 81%). However, Yeh et al. [21] reported no significant advantage of high-dose PPI–amoxicillin dual therapy in either ITT or PP analyses (OR 0.92 and 0.88, respectively). Conversely, a meta-analysis by Li et al. [22] suggested that administering amoxicillin four times daily might improve efficacy. Vonoprazan, as a potassium-competitive acid blocker (P-CAB), offers a novel approach to dual therapy owing to its rapid and sustained acid-suppressive effects.

Since its introduction to the market, there have been numerous studies on vonoprazan-based dual therapy, but several issues remain. Previous research has primarily focused on vonoprazan-high-dose amoxicillin dual therapy [23,24,25,26,27,28,29,30,31], including both RCTs and real-world studies. ITT analyses showed eradication rates ranging from 78.5% to 96.0%, while PP analyses demonstrated eradication rates between 81.2% and 97.9%. However, some studies [32,33] have found that 7-day or 10-day courses of vonoprazan high-dose amoxicillin yielded suboptimal efficacy. Therefore, no consensus has been reached regarding the optimal dosage and frequency of amoxicillin administration. Additionally, the potential side effects of this dual regimen remain unknown. In this trial, we deliberately selected a low daily dose of amoxicillin (2 g/day) for the vonoprazan–amoxicillin dual therapy regimen. The rationale was twofold. Amoxicillin maintains a very low global resistance rate (approximately 6%), and pharmacodynamic studies have suggested that even moderate dosing can achieve sufficient bactericidal concentrations in the gastric mucosa when combined with potent and sustained acid suppression by vonoprazan.

The study preliminarily evaluated the efficacy and safety of vonoprazan low-dose amoxicillin (2 g/day) through a randomized controlled clinical trial involving 504 patients. The results demonstrated that the vonoprazan low-dose amoxicillin dual regimen was non-inferior to traditional bismuth-containing quadruple therapy in treatment-naïve *H*. *pylori* patients, with a PP eradication rate exceeding 90%, meeting the ‘good’ standard according to the REGARD criteria [34]. Through multi-dimensional analyses (ITT, mITT, and PP), it consistently confirmed no significant difference in *H. pylori* eradication rates between the VA and EBAC groups (*p* > 0.05), with the VA group satisfying the non-inferiority criteria. Although the ITT analysis showed a borderline statistical trend (*p* = 0.060), the absolute difference (6%) and substantial overlap in the 95% CI suggested limited clinical significance. Thus, the VA regimen remains a robust non-inferior alternative. This marginal discrepancy might stem from the inclusion of patients who did not complete treatment (e.g., lost to follow-up), potentially leading to underestimation. Further stringent PP and mITT analyses revealed negligible differences (<1%), reinforcing the robustness of the findings. These results provide rigorous evidence-based support for the VA regimen and strengthen the reliability of the conclusions.

Safety evaluation demonstrated that the VA group had significantly fewer TEAEs than the EBAC group (27.2% vs. 42.7%, *p* < 0.001). This difference was primarily driven by EBAC’s higher incidence of clarithromycin-associated dysgeusia (69 cases vs. 2 cases in VA). While abdominal discomfort (28 vs. 16 cases) and hunger sensation (8 vs. 2 cases) were more frequently reported in the VA group—potentially related to proton pump inhibition by P-CABs—both groups showed comparable rates of moderate-to-severe adverse events (<10% each). Discontinuation rates due to AEs showed no statistical difference (VA 1.6% vs. EBAC 2.0%, *p* = 0.737), with rash being the main reason in EBAC versus abdominal discomfort, vomiting/diarrhea, and lower limb weakness in VA. All symptoms resolved after discontinuation, indicating good overall tolerability for both regimens. Although medication compliance was numerically higher in EBAC (96.3% vs. 93.5%), this difference lacked both clinical and statistical significance (*p* = 0.151), further supporting VA’s clinical feasibility.

The VA regimen also demonstrated superior cost-effectiveness, with medication expenses approximately half of EBAC (330.50 CNY versus 675.68 CNY). This economic advantage, combined with its simplified dosing and favorable safety, supports VA dual therapy as a practical first-line option—particularly for patients with comorbidities, contraindications to bismuth, or intolerance to clarithromycin-based regimens.

Based on these findings, the VA regimen demonstrates superior safety (with significantly lower TEAE incidence) and patient-friendliness (featuring simplified dosing and lower cost) while maintaining non-inferior efficacy, making it a viable first-line option for *H. pylori* eradication—particularly suitable for patients with comorbidities (e.g., mild hepatic/renal impairment or bismuth contraindications), those requiring simplified regimens (e.g., busy professionals or patients with history of drug intolerance like dysgeusia/nausea), and cases where traditional quadruple therapy proves challenging. Future studies should further explore its cost-effectiveness in specific populations including elderly patients and high antibiotic-resistance regions, to better define its role in clinical practice.

This study employed logistic regression analysis to evaluate potential factors influencing eradication rates, revealing medication adherence as the sole statistically significant independent predictor (*p* < 0.001). Demographic characteristics including gender, age, BMI, and education level showed no significant association. This finding underscores the critical importance of enhanced medication management in clinical practice. Notably, international studies have demonstrated geographical variations in VA dual therapy efficacy, with superior performance observed in Asian populations compared to Western and Taiwanese cohorts [20,30,35]. This discrepancy likely results from multiple interacting factors [36]: (1) differences in antibiotic dosing regimens, (2) regional variations in *H. pylori* resistance patterns, (3) disparities in gastric pH control (particularly concerning CYP2C19 rapid metabolizer distribution), and (4) host characteristics such as body weight. The cohort’s median BMI of 24.2 was significantly lower than Western studies (28.7–29.1), and this reduced BMI may enhance efficacy through pharmacokinetic mechanisms like altered drug distribution volume. Interestingly, the bismuth-containing quadruple therapy demonstrated higher efficacy (92.7% mITT, 93.0% PP) than previous reports [9,24,33,36]. Beyond BMI differences, this was attributed to two distinctive cohort characteristics: first, the high proportion of highly educated participants (>70% college graduates in both groups) potentially improving treatment comprehension and execution; second, excellent adherence rates (>90% in both arms), directly validating adherence as the key predictive factor in the univariate and multivariate analyses. These observations emphasize the necessity of considering sociodemographic and behavioral factors when interpreting inter-study efficacy variations.

This study has several limitations that should be acknowledged. First, as an open-label, non-double-blind trial, it may have introduced reporting bias, particularly regarding adverse events, and thus should be interpreted with caution. Second, being conducted at a single center, the findings require validation through multicenter studies to confirm generalizability to broader populations. Third, it is important to emphasize that the success of VA dual therapy has been primarily demonstrated in Asian populations. Data supporting its efficacy in Western countries remain limited, where differences in antibiotic resistance patterns (notably higher rates of clarithromycin and metronidazole resistance) and a lower prevalence of CYP2C19 loss-of-function polymorphisms may significantly impact treatment outcomes. Fourth, the limited availability of vonoprazan in many Western countries (only approved in the US in 2022) may restrict the therapy’s widespread adoption. Fifth, this study did not systematically collect data on participants’ socioeconomic status, which may be an important factor influencing treatment adherence and outcomes. Future prospective studies should consider incorporating this variable into their design. Sixth, VA regimen cannot serve as an alternative for patients with penicillin allergies. A key limitation of this study is the lack of data on antibiotic resistance profiles of *H. pylori*, which may affect the interpretation of the treatment outcomes and their generalizability. Despite these limitations, the findings provide valuable evidence supporting VA dual therapy as a first-line option for *H. pylori* treatment-naïve patients. Future multicenter, resistance-guided, and geographically diverse trials are warranted to further establish the optimal amoxicillin dosing schedule, validate cost-effectiveness in special populations, and confirm the generalizability of findings outside Asia.

## 5. Conclusions

The 14-day vonoprazan low-dose amoxicillin dual therapy demonstrated non-inferior efficacy and significantly improved safety compared to bismuth-containing quadruple therapy in treatment-naïve *H. pylori* patients. Given its favorable safety profile, simplified administration, and lower cost, the VA regimen represents a promising first-line option, particularly in settings with high clarithromycin resistance, elderly patients, or in patients intolerant to complex regimens. Future multicenter, double-blind studies are warranted to further optimize dosing strategies and confirm generalizability across diverse populations.

## Figures and Tables

**Table 1:** Baseline characteristics of patients.

	EBAC (n = 252)	VA (n = 252)		*p*
Age (years, Median (P75–P25))	40.00 (50.75, 34.00)	40.00 (52.00, 32.00)	−0.919	0.358
			0.143	0.931
<45	159 (63.1%)	160 (63.5%)		
45–59	65 (25.8%)	62 (24.6%)		
≥60	28 (11.1%)	30 (11.9%)		
Gender (male, n%)	133 (51.8%)	119 (47.2%)	0.032	0.858
BMI (Median (P75–P25))	24.20 (26.20, 22.03)	24.20 (26.40, 21.50)	−0.580	0.562
			1.174	0.759
<18.5	8 (3.2%)	10 (4.0%)		
18.5–23.9	106 (42.1%)	111 (44.0%)		
24.0–27.9	104 (41.3%)	93 (36.9%)		
≥28.0	34 (13.5%)	38 (15.1%)		
Cigarette smoking	40 (15.9%)	44 (17.5%)	3.193	0.203
Alcohol drinking	70 (27.8%)	64 (25.4%)	0.366	0.545
Family history of gastric cancer	10 (4.1%)	6 (2.4%)	1.033	0.310
Symptoms			11.010	0.201
No symptoms	95 (37.7%)	82 (32.5%)		
Acid reflux or heart burn	59 (23.4%)	89 (35.3%)		
Distention	44 (17.5%)	53 (21.0%)		
Abdominal pain	35 (13.9%)	38 (15.1%)		
Halitosis	24 (9.5%)	21 (8.3%)		
Belching	24 (9.5%)	19 (7.5%)		
Nausea	11 (4.4%)	13 (5.2%)		
Abdominal discomfort	7 (2.8%)	11 (4.4%)		
Others	6 (2.4%)	2 (0.8%)		
Diagnosis with endoscopy			2.330	0.312
Chronic gastritis	96 (38.1%)	111 (44.0%)		
Peptic ulcer	9 (3.6%)	11 (4.4%)		
No endoscopy	147 (58.3%)	130 (51.6%)		
Chronic disease			4.926	0.295
Hypertension	28 (11.1%)	31 (12.3%)		
Diabetes mellitus	9 (3.6%)	13 (5.2%)		
Cardiovascular or cerebrovascular diseases	4 (1.6%)	9 (3.6%)		
Renal insufficiency	1 (0.4%)	1 (0.4%)		
Others	5 (2.0%)	2 (0.8%)		

EBAC, esomeprazole, bismuth, amoxicillin, and clarithromycin; VA, vonoprazan, amoxicillin; BMI, body mass index.

**Table 2:** Comparison of eradication rates between EBAC and VA groups.

Eradication Rates	EBAC Group	VA Group	*p*	Rate Difference (95% Cl)
ITT	85.7% (216/252)	79.4% (200/252)	0.060	0.0635
95% CI	[81.4%, 90.1%]	[74.3%, 84.4%]		[−0.58%, 13.23%]
mITT	92.7% (216/233)	91.7% (200/218)	0.714	0.0096
95% CI	[89.3%, 96.1%]	[88.1%, 95.4%]		[−4.39%, 6.45%]
PP	93.0% (213/229)	92.1% (197/214)	0.712	0.0096
95% CI	[89.7%, 96.3%]	[88.4%, 95.7%]		[−4.36%, 6.42%]

EBAC, esomeprazole, bismuth, amoxicillin, and clarithromycin; VA, vonoprazan, amoxicillin; ITT, intention-to-treat; mITT, modified intention-to-treat; PP, per-protocol.

**Table 3:** Logistic univariate and multivariate analysis of factors affecting eradication rate.

	Multivariate Analysis		
	OR	95% CI	*p*
Age (years)			0.650
<45	1		
45–59	1.227	(0.435–3.466)	0.699
≥60	0.844	(0.313–2.277)	0.738
BMI			0.610
<18.5	1		
18.5–23.9	1.080	(0.195–5.987)	0.930
24.0–27.9	1.067	(0.427–2.668)	0.889
≥28.0	1.553	(0.645–3.741)	0.326
Gender			
male	1		
female	1.372	(0.724–2.602)	0.332
Cigarette smoking			
no	1		
yes	2.224	(0.855–5.783)	0.101
Alcohol drinking			
no	1		
yes	1.181	(0.565–2.471)	0.658
Family history of gastric cancer			
no	1		
yes	0.442	(0.114–1.706)	0.236
Diagnosis with endoscopy			0.960
No endoscopy	1		
Chronic gastritis	1.182	(0.279–5.000)	0.820
Peptic ulcer	1.109	(0.262–4.693)	0.889
Adherence			
<80%	1		
≥80%	17.557	(5.678–54.287)	<0.001
Group			
EBAC group	1		
VA group	0.579	(0.330–1.013)	0.055

EBAC, esomeprazole, bismuth, amoxicillin, and clarithromycin; VA, vonoprazan, amoxicillin; BMI, body mass index.

**Table 4:** Adverse events in each group.

	EBAC (n = 246)	VA (n = 246)	*p*
Total, n/N (%)	105/246 (42.7%)	67/246 (27.2%)	<0.001
AE grade			0.767
Mild	96 (91.4%)	59 (88.1%)	
Moderate	8 (7.6%)	7 (10.4%)	
Severe	1 (1.0%)	1 (1.5%)	
AE variety			
Bitter taste	69	2	
Diarrhea	19	13	
Abdominal discomfort	16	28	
Nausea	16	10	
Rash	9	5	
Vomiting	7	1	
Hunger sensation	2	8	
Dizziness and headache	1	4	
Decreased appetite	1	3	
Hyperhidrosis	1	1	
Tinnitus	1	0	
Constipation	0	6	
Pharyngeal discomfort	0	3	
Arthralgia and weakness	0	2	
Insomnia	0	1	
Discontinued due to AEs	5/246 (2.0%)	4/246 (1.6%)	0.737
Adherence, n/N (%)	237/246 (96.3%)	230/246 (93.5%)	0.151

AE, adverse event; EBAC, esomeprazole, bismuth, amoxicillin, clarithromycin; VA, vonoprazan and amoxicillin.

## Data Availability

Data available on request due to privacy/ethical restrictions.

## Registration Number

chictr.org.cn, ChiCTR2500098029.
